# Promising Combinatorial Therapeutic Strategies against Non-Small Cell Lung Cancer

**DOI:** 10.3390/cancers16122205

**Published:** 2024-06-12

**Authors:** Prabhjot Kaur, Santosh Kumar Singh, Manoj K. Mishra, Shailesh Singh, Rajesh Singh

**Affiliations:** 1 Department of Microbiology, Biochemistry, and Immunology, Morehouse School of Medicine, Atlanta, GA 30310, USA; prakaur@msm.edu (P.K.); sksingh@msm.edu (S.K.S.); shsingh@msm.edu (S.S.); 2 Cancer Biology Research and Training, Department of Biological Sciences, Alabama State University, Montgomery, AL 36014, USA; mmishra@alasu.edu; 3 Cancer Health Equity Institute, Morehouse School of Medicine, Atlanta, GA 30310, USA

**Keywords:** tyrosine kinase inhibitors, clinical trials, immune checkpoint inhibitors, PARP inhibitors, PDL-1, nanobodies, natural drug therapy

## Abstract

**Simple Summary:**

Lung cancer treatment options vary depending on the type and stage of the cancer. For non-small cell lung cancer (NSCLC), treatments may include surgery, chemotherapy, radiation therapy, targeted therapy, or a combination of these methods. Targeted therapies have demonstrated efficacy as adjuvant treatment in early-stage NSCLC when used alongside primary interventions such as surgery. In advanced stages of NSCLC, targeted therapies serve as a means of palliative care, enhancing quality of life and extending survival time. Our review article focuses on recent evidence of various treatment approaches and their impact on overall and progression-free survival in NSCLC patients, particularly targeting specific molecular and genetic alterations associated with NSCLC.

**Abstract:**

Non-small cell lung cancer (NSCLC) presents a complex and diverse disease, exhibiting variations at individuals’ cellular and histological levels. This complexity gives rise to different subtypes and genetic mutations, posing challenges for accurate diagnosis and effective treatment. Nevertheless, continuous progress in medical research and therapies is continually shaping the landscape of NSCLC diagnosis and management. The treatment of NSCLC has undergone significant advancements in recent years, especially with the emergence of targeted therapies that have shown remarkable efficacy in patients with actionable mutations. This has ushered in the era of personalized medicine in NSCLC treatment, with improvements in molecular and immunohistochemical techniques contributing to enhanced progression-free survival. This review focuses on the latest progress, challenges, and future directions in developing targeted therapies for NSCLC, including tyrosine kinase inhibitors (TKIs), DNA-damaging agents, immunotherapy regimens, natural drug therapy, and nanobodies. Furthermore, recent randomized studies have demonstrated enhanced overall survival in patients receiving different targeted and natural drug therapies.

## 1. Introduction

Non-small cell lung carcinoma (NSCLC) is the most prevalent and severe type of cancer, comprising up to 85% of all lung malignancies. Unfortunately, NSCLC is a particularly resistant type of cancer, and it frequently metastasizes to the brain, leading to a 5-year survival rate of 15%. NSCLC is histologically classified into three major subtypes, i.e., lung adenocarcinoma (LUAD), squamous cell carcinoma (SQCC), and large cell lung carcinoma (LCLC). Among all, LUAD is the most common cancer type in non-smokers and represents 40% of all lung cancers. The second subtype, SQCC, is predominantly found in men and diagnosed during advanced stages of cancer, accounting for 25–30% of all lung cancers. On the other hand, the third subtype, LCLC, comprises around 10% of all cases. According to the North American Association of Central Cancer Registries, lung and bronchus cancer results in an estimated 127,070 deaths and 238,340 new cases annually. While incidence trends favor men, they have decreased in women by half [1].

**Figure 1:**
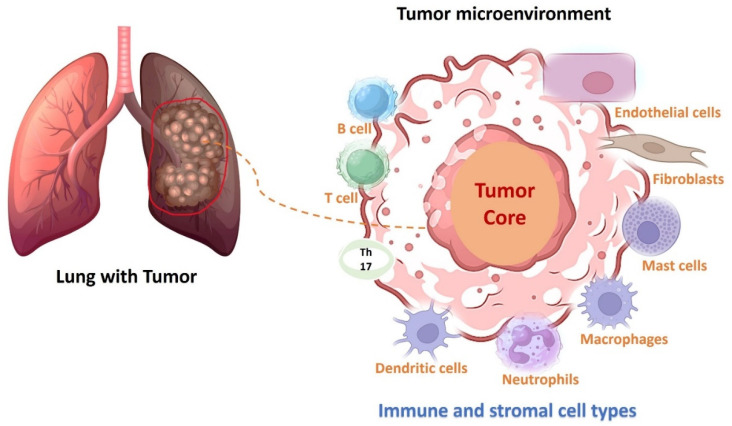
Intratumoral heterogeneity in non-small cell lung cancer (NSCLC) at the advanced stage demonstrates different tumor microenvironment cell types.

Chemotherapeutic drugs, viz., carboplatin, cisplatin, docetaxel, Paclitaxel, gemcitabine, and pemetrexed, are commonly used in primary cancer treatment. However, lung cancer survivors often experience physical and emotional effects that persist long after treatment. Unfortunately, chemotherapy can cause side effects and off-target toxicity in healthy tissues. Over 90% of patients experience nausea and vomiting, requiring anti-emetic medication during treatment [2]. Although platinum-based chemotherapy has been the primary treatment at the advanced stage, novel strategies for managing NSCLC have recently been developed, including checkpoint inhibition, immunomodulation, and actionable oncogenic driver mutations. Therapeutic decisions for managing NSCLC should be based on the molecular characteristics of tumor tissues. NSCLC, at its advanced stage, is characterized by a large heterogeneity, demonstrating the presence of various immune cells, tumor-infiltrating myeloid cells (TIMs), and stromal cells (Figure 1). Overall, targeted therapy with tyrosine kinase inhibitors (TKIs) and immunotherapy with immune checkpoint inhibitors have significantly improved the survival rate of NSCLC patients at advanced or metastatic stages. In recent years, the Food and Drug Administration (FDA) has approved several TKIs and monoclonal antibodies that target PD-1 and PD-L1 as first-line treatment options for advanced NSCLC [3].

Understanding the genetic alterations or mutations that give rise to NSCLC is an ever-evolving field. The approach of targeted therapy and precision medicine has drastically transformed the way cancer is approached. These advances have benefited patients whose disease was otherwise considered incurable at metastatic or advanced stages. However, despite the potential these therapies hold, resistance to single drugs often leads to treatment failure, and only a small number of patients experience long-term benefits. This review highlights the promising strategies to overcome resistance with the combination of drugs. Recent advancements in personalized and targeted therapies are gaining prominence, offering hope for NSCLC patients continually developing acquired resistance. Exploring the emergence of secondary mutations in cancer cells and the development of next-generation inhibitors holds the potential to effectively address drug resistance, particularly when there is a comprehensive understanding of the molecular mechanisms associated with resistance. These interventions aim to improve treatment efficacy and patient outcomes by targeting specific molecular and genetic alterations associated with NSCLC. Addressing the clinical challenges in developing these combinations and considering future perspectives will be crucial in optimizing cancer treatment strategies. This review aims to provide an overview of the complexities associated with these emerging treatments and the latest therapeutic approaches and trends used to manage NSCLC disease.

## 2. Targeted Therapies

Precision oncology, which relies on actionable molecular markers, is now the evidence-based standard of care that has brought light to managing advanced NSCLC. Personalized therapy has transformed the therapeutic landscape by expanding treatment options for advanced NSCLC patients through targeted therapies based on genetic and epigenetic cues. In this section, we have discussed the FDA-approved therapeutic progress of targeted therapies and their possible combination therapy in pre-clinical and clinical studies.

The discoveries of tumor-driver genes such as epidermal growth factor receptor (EGFR), anaplastic lymphoma kinase (ALK), mesenchymal-epithelial transition (MET), and c-ros oncogene 1 (ROS1) in NSCLC have recommended TKIs as the first-line option for patients with advanced stage disease.

### 2.1. Epidermal Growth Factor Receptor (EGFR) Inhibitors

The EGFR is a cell surface receptor that plays a key role in cancer cell proliferation, differentiation, and migration. EGFR comprises two domains—an extracellular ligand-binding domain and an intracellular tyrosine kinase domain. The intracellular domain gets activated by binding ligands to the extracellular domain of EGFR. Upon ligand binding, EGFR undergoes dimer formation and auto-phosphorylation at the tyrosine residues, which is crucial in activating downstream signaling pathways such as MAPK AKT, JAK/STAT, and PI3K [4]. Overexpression and mutation of EGFR are associated with poor prognosis and tumor progression in non-small cell lung cancer (NSCLC). Approximately 20% of NSCLC patients have activating mutations in the EGFR. The deletions in exon 19 (*Ex19del*) and the *L858R* point mutation within exon 21 make up around 90% of EGFR-activating mutations. Targeting EGFR for therapeutic intervention is a rational approach for developing novel anticancer agents. In European patients, EGFR mutations account for approximately 14% of mutations, while in North and South America, they make up 25% of mutations. Meanwhile, Asian patients have the highest percentage of these mutations, with 40% [5].

In treating metastatic NSCLC patients, TKIs targeting the EGF receptor have been used for the past decade. These TKIs are effective in targeting oncogene-driven activating EGFR mutations. Since the first approval by the FDA in 2003, EGFR-TKIs have achieved remarkable success for advanced NSCLC patients. Over the past two decades, three generations of EGFR-TKIs showed efficacy in clinical trials. The first-generation EGFR-TKI drugs, including gefitinib (FDA approved, 2003), erlotinib (FDA approved, 2004), and icotinib, are reversible and ATP-competent inhibitors that showed promising results when used in the first-line treatment for NSCLC patients harboring EGFR mutations. These drugs have demonstrated better survival rates and median progression-free survival (PFS) than first-line platinum-based doublet chemotherapy. Second-generation EGFR-TKIs such as afatinib (approved by FDA in 2013) and dacomitinib (approved by FDA in 2018), along with third-generation EGFR-TKIs like osimertinib (approved by FDA in 2015), furmonertinib, lazertinib, and rezivertinib, are irreversible inhibitors [6]. A third-generation inhibitor, osimertinib, is a potent and selective inhibitor of both EGFR-activating mutations and *EGFR-T790M*. However, recent studies reported EAI045 [7] and BLU-945 [8] as potent next-generation (fourth-generation) novel EGFR TKIs that mediate the resistance to third-generation inhibitors for EGFR mutant-driven NSCLC targeting *T790M* and *C797S* mutations.

#### 2.1.1. First-, Second-, and Third-Line EGFR Inhibitors with Combination Therapy Setting

In recent studies, it has been shown that simultaneously targeting EGFR (epidermal growth factor receptor) and VEGFR (vascular endothelial growth factor receptor) signaling can be an effective first-line treatment approach, particularly in addressing resistance to anti-EGFR therapies with Exon 19 deletion or *L858R* point mutations. Clinical trials have demonstrated the potential efficacy of combining EGFR and VEGFR therapies in improving outcomes for NSCLC patients with EGFR mutations. For instance, the addition of bevacizumab (an anti-VEGF antibody) to gefitinib [9] and erlotinib [10,11] has shown promising results in treating NSCLC. However, it’s important to note that these combination therapies may be associated with common adverse effects. In a specific clinical trial involving treatment-naive NSCLC patients with advanced EGFR mutations, the combined use of apatinib (an oral angiogenesis inhibitor targeting VEGFR2) and gefitinib as a first-line treatment has been found to improve progression-free survival (PFS) [12]. This combination treatment also resulted in a higher incidence of treatment-emergent adverse events when compared to the placebo and gefitinib treatment.

The first allosteric inhibitor that targets the EGFR mutants (*T790M* and *C797S*), EAI045, has shown effectiveness in combination with cetuximab (a monoclonal antibody) [13]. However, further clinical trials are essential to confirm its efficacy in treating patients with advanced NSCLC. NCT04862780, an active, open-phase 1/2 clinical study, involved conducting a first-in-human (FIH) to address acquired resistance mechanisms in patients with EGFR mutants (*T790M* and *C797S* mutation). Its primary objective was to evaluate the safety, tolerance, pharmacokinetics (PK), pharmacodynamics (PD), and anti-cancer effects of BLU-945, explicitly targeting the EGFR protein. The study assessed the use of this inhibitor as a monotherapy treatment and in combination with osimertinib. In recent randomized phase II trials, researchers have found that combining gefitinib and pemetrexed holds promise in addressing acquired resistance to EGFR inhibitors. The results indicate a significantly longer progression-free survival (PFS) compared to gefitinib monotherapy [14]. Furthermore, a separate study suggests that Icotinib may be as effective as gefitinib and less toxic as a first-line treatment for advanced EGFR mutation-positive patients compared to other chemotherapeutic regimens such as cisplatin/pemetrexed plus pemetrexed [15]. Similarly, afatinib has demonstrated good efficacy with manageable toxicity in patients with lung adenocarcinoma positive for EGFR-activating mutations [16]. This study suggests that by following guidelines for managing adverse events and reducing the initial dosage of afatinib, severe toxicities could be mitigated. Additionally, Mok et al. [17] reported significant improvement in progression-free survival with dacomitinib compared to the standard of care TKI-gefitinib. Further details on clinical trials involving all EGFR TKIs in NSCLC patients with different gene mutations have been discussed in Table 1.

In November 2015, the FDA granted approval for the use of osimertinib for treating NSCLC patients with the acquired *T790M* mutation. One latest study has raised concerns about the potential risk of interstitial lung disease (ILD) when osimertinib is combined with durvalumab, an immune checkpoint inhibitor (ICI). Therefore, caution is advised in further exploring the combination of EGFR TKIs with ICIs [18]. A recent study focusing on patients with advanced NSCLC and EGFR *T790M* mutation found promising results with a new third-generation EGFR tyrosine kinase inhibitor called rezivertinib [19]. Another newly designed pan-EGFR TKI, furmonertinib, showed significant improvement in patients with specific NSCLC mutations and CNS metastases compared to gefitinib as a first-line treatment [20]. Lazertinib, a potent third-generation EGFR TKI, has also demonstrated enhanced efficacy in treating brain metastases in *T790M*-positive patients compared to Osimertinib [21].

The approach to treating EGFR exon 19 deletion, *L858R* point mutation, and *T790M* mutation has advanced personalized drug therapy based on tumor genotyping, moving away from a one-size-fits-all approach. Initial treatment now involves using anti-EGFR agents and combining VEGFR inhibitors (such as bevacizumab and apatinib) with targeted therapy for patients with advanced NSCLC. This allows for managing acquired resistance to EGFR mutation-targeted therapies.

**Table 1:** EGFR tyrosine kinase inhibitors with combination therapy/agent in NSCLC.

Inhibitors for EGFR Rearrangements and Combination Therapy	Starting Dose	Stage of Disease	Previous Treatment	Sample Size (Phase)	Objective Response Rate (ORR) and/or Median Progression-Free Survival (PFS)	References
**Exon 19 deletion or *L858R* point mutation**						
Gefitinib + Bevacizumab	Gefitinib (250 mg/day) and bevacizumab (15 mg/kg, every 3 weeks)	Stage IIIB or IV	Untreated	42 patients (phase 2)	ORR: 73.8%, PFS: 12 months	[9]
Erlotinib + Bevacizumab	Erlotinib (150 mg/day) and bevacizumab (15 mg/kg) every 21 days	Advanced	Not reported	160 patients (phase 3)	PFS: 13.8 months	[10]
	Erlotinib (150 mg/day) and bevacizumab (900.0 mg/cycle)-22 cycles	Stage IIIB or IV	Untreated	311 patients (phase 3)	ORR: 86.8%, PFS:17.9 months	[11]
Gefitinib + Apatinib	Gefitinib (250 mg/day) and Apatinib (500 mg/day)	Stage IIIB or IV	Not reported	313 patients (phase 2)	ORR: 77.1%, PFS: 13.7 months	[12]
Gefintib + Pemetrexed	Gefintib (250 mg/day) and Pemetrexed (500 mg/m^2^, 3-weekly)	Stage IV	Untreated	196 patients (phase 2)	PFS: 16.2 months	[14]
Icotinib	(125 mg/day) 3 times per day	Stage IIIB or IV	Untreated	270 patients (phase 3)	PFS: 11.2 months	[15]
Afatinib	20–40 mg/day	Stage IIIB or IV	Untreated with EGFR inhibitors	35 patients	ORR: 77.1%, PFS: 13.8 months	[16]
Dacomitinib	45 mg/day	Advanced	Untreated	227 patients (phase 3)	ORR: 74.9%, PFS: 14.7 months	[17]
**Exon 19 deletion or *L858R* point mutation and *T790M* mutation**						
Osimertinib	80 mg/day	Advanced	Untreated	52 patients in one arm	PFS: 18 months	[22]
Osimertinib + Durvalumab	Osimertinib (80 mg/day) and durvalumab (3 or 10 mg/kg), every 2 weeks	Advanced	Part A: pre-treated with EGFR inhibitors and Part B: untreated	34 patients (phase 1b)	Part A: ORR 43%. Part B: ORR 82%	[18]
Rezivertinib	30 mg	Advanced	Untreated	153 patients (phase 1)	PFS: 9.7 months	[19]
Furmonertinib	80 mg/day	Advanced	Untreated except 4% of patients	133 patients with CNS lesions	CNS-ORR: 91% CNS-PFS: 20.8 months	[20]
Lazertinib	240 mg/day	Advanced	Pre-treated with EGFR-TKIs	78 patients (phase 1/2)	ORR: 55.3%, PFS: 11.1 months	[21]

#### 2.1.2. Combination of EGFR Inhibitors with Anticancer Drugs Targeting Epigenetic Factors

Enduring gene changes, including point mutations, deletions, translocations, amplifications, and epigenetic modifications, drive the development and advancement of lung cancer. These alterations impact various processes, including histone modifications, DNA methylation, and microRNA regulation. Increased expression of histone deacetylases (HDACs) is a common feature in many cancers, including lung cancer, and HDAC inhibitors show great potential in their treatment. HDAC inhibitors influence gene transcription and can block angiogenesis by inhibiting factors like vascular endothelial growth factor (VEGF) [23].

Research shows that combining HDAC inhibitors (HDACis) with cancer treatments, such as EGFR-TKI, has proven more effective in several settings. HDACis and TKIs impact similar downstream pathways, influencing the expression of cell cycle regulators like p21, p53, and CHK1 expression. Reduced CHK1 levels may be a biomarker for HDACi response in NSCLC patients, associated with decreased E-cadherin expression. E-cadherin depletion leads to cancer metastasis and resistance to TKI treatment. HDACis can enhance E-cadherin expression, making it a positive prognostic marker for lung cancer and a potential indicator of TKI response. Well-tolerated combination therapies of HDACis and TKIs have shown effectiveness, particularly in patients with high E-cadherin expression. In a recent phase II study, the combination of erlotinib with entinostat, a selective HDAC inhibitor, showed promising results in prolonging progression-free survival in non-small cell lung cancer (NSCLC) patients with high E-cadherin levels, irrespective of their EGFR genotype [24]. In preclinical studies, combining panobinostat (an HDAC inhibitor) and erlotinib has increased the antiproliferative effect on EGFR-mutant and wild-type cell lines. This combination affects the expression of downstream targets of the EGFR pathway. Erlotinib has been shown to enhance panobinostat-induced acetylation of histone H3 [25]. In another preclinical study, the combination of LBH589 (panobinostat) and osimertinib demonstrated a synergistic effect in reducing the survival of different osimertinib-resistant cell lines, including those harboring *C797S* mutations in NSCLC [26]. Additionally, researchers conducted preclinical studies to investigate the in vitro and in vivo inhibitory effects of bromodomain and extra-terminal proteins (BET) inhibitors as well as HDAC inhibitors, trichostatin A (TSA), and vorinostat on tumor growth. They also studied the expression of BET proteins in osimertinib-sensitive and osimertinib-resistant cell lines. These studies found that bromodomain and extra-terminal proteins (BETs) were upregulated in osimertinib-resistant cells compared with those in the paired parental cells and that knockdown of BETs significantly inhibited the growth of osimertinib-resistant cells. The findings from these studies may lead to the development of new treatment targets and strategies for non-small cell lung cancer resistant to osimertinib [27]. The BET proteins play a crucial role as “readers” of DNA. They are involved in regulating the growth of cells through epigenetic mechanisms. BET proteins work by binding to acetylated histones. This binding process leads to the activation of gene promoters, ultimately facilitating the transcription of oncogenes.

In *EGFR*-mutated NSCLC patients, a genetic variation known as BIM deletion has been linked to poor responses to EGFR TKI. A phase I study found that combining vorinostat with gefitinib led to increased acetylated histone H3 protein expression levels and decreased *BIM* mRNA exon 3/exon 4 ratio in PBMC. These findings suggest that the combination treatment could potentially counteract the negative effects of the BIM deletion, which is a common genetic variant associated with resistance to EGFR-targeted therapy in NSCLC with EGFR mutations [28].

### 2.2. Inhibitors for ALK Rearrangements

The Anaplastic lymphoma kinase-ALK (*CD246*) gene encodes a transmembrane receptor tyrosine kinase that helps in normal cell proliferation and neurogenesis. Echinoderm microtubule-associated proteins like 4 (EML4)-ALK rearrangements were first discovered in 2007, for NSCLC patients, and later, many EML4 breakpoints were identified. ALK rearrangements occur in about 5–8% of adenocarcinoma cases but are responsive to ALK inhibitors in various clinical trials, improving the median progression-free survival in patients. Crizotinib, a first-generation inhibitor, is a target of p-glycoprotein and can lead to brain relapse. However, patients with ALK-positive NSCLC can quickly develop brain metastasis. Consequently, 2nd and 3rd generation ALK inhibitors that can cross the blood-brain barrier and show marked intracranial anti-tumor activity in advanced ALK-positive patients have been developed [4].

#### First, Second-, and Third-Line ALK Inhibitors with Combination Therapy Setting

In first-line treatment studies, ceritinib [29] and crizotinib [30] demonstrated a significant and clinically meaningful improvement in PFS compared to platinum-based chemotherapy. As per recent reports, alectinib significantly delayed CNS progression versus crizotinib [31] and is also a preferred first-line treatment with advanced ALK-positive NSCLC that is approved for the treatment of patients who are intolerant to crizotinib [32]. The first-generation drugs ceritinib and alectinib, as well as the second-generation drug brigatinib, have been approved as second-line treatments for advanced or metastatic NSCLC and have shown strong effectiveness in Table 2. Additionally, a novel third-generation TKI called lorlatinib [33], as well as brigatinib [34], have displayed higher progression-free survival rates in clinical trials compared to crizotinib in phase III trials for advanced ALK-positive patients. Lorlatinib, an important novel strategy, exhibited a disease control rate of 91% in advanced NSCLC patients with ALK/ROS1 rearrangements who failed to respond to first- and second-generation TKIs [33]. Lin et al. [35] reported the dose-limiting toxicities and safety of adding bevacizumab (an anti-VEGF inhibitor) to the alectinib inhibitor in a dual combination of ALK-VEGF.

Many patients with ALK-positive NSCLC initially respond well to the drug crizotinib. However, the positive effects are often short-lived due to the development of acquired resistance. Acquired resistance is a significant challenge for ALK inhibitors and targeted therapies. In some cases, secondary mutations have been found in the EML4-ALK gene within tumor cells from patients who relapsed after ALK inhibitor treatment [36]. These mutations, occurring independently within the tumor, significantly reduce the effectiveness of different ALK inhibitors. Such resistance mechanisms are broadly categorized as on-target genetic alterations (e.g., ALK resistance mutations, ALK gene amplification) or off-target mechanisms (e.g., activation of alternative signaling pathways). Interestingly, these mechanisms can coexist in the same cancer, suggesting that a combination of ALK and EGFR inhibitors could potentially be an effective treatment for certain NSCLC patient subsets [37]. While TKIs have shown promise as a first-line option for brain metastases in NSCLC-mutated patients, some individuals develop resistance and experience relapse. Therefore, exploring the underlying resistance mechanisms and developing combination drug therapies to reverse this resistance is essential.

### 2.3. Inhibitors for ROS1 Fusion Variants and Combination Therapy

ROS1 (C-ros oncogene 1) is an intracellular tyrosine kinase receptor known to have over 20 ROS1 gene rearrangements or fusions in ~2% of NSCLC cases, with *CD74-ROS1* being the most common variant (38–54%). ROS-1-positive patients also have exclusive co-mutation with EFGR, ALK, and KRAS gene rearrangements [38]. Targeted therapy with Crizotinib was approved for the first time worldwide for ROS1 fusion tumors. However, several studies have documented that central nervous system (CNS) metastases developed in approximately 40% of the patients after treatment with ROS-1 fusion inhibitors [39]. New generation inhibitors (ensartinib, entrectinib, lorlatinib, repotrectinib, and taletrectinib) have been discovered after the development of resistance to crizotinib in NSCLC patients (Table 3), and they have better brain penetration [40]. Entrectinib is now approved in the United States, Europe, and Japan.

The FDA has approved crizotinib as a treatment for NSCLC with certain genetic mutations. This drug targets ALK, ROS1, and MET rearrangements and is still considered the first-line therapy [38,41]. Whereas ensartinib has demonstrated moderate efficacy in treating non-small cell lung cancer NSCLC with ROS1 mutations. It has also shown a favorable safety profile, particularly in cases of central nervous system (CNS) progression following prior treatment with crizotinib [42]. Nonetheless, there are still gaps in the treatment of NSCLC, including the need to tackle resistance mutations, enhance efficacy in treating brain metastases, and reduce neurological side effects. A newly developed targeted therapy named taletrectinib aims to meet these needs by offering improved efficacy, overcoming resistance to first-generation ROS1 inhibitors, and targeting brain metastases while causing fewer neurological adverse events [43].

#### First, Second-, and Third-Line ROS1 Inhibitors with Combination Therapy Setting

The effectiveness of various ROS1 inhibitors has been studied extensively. These multi-kinase inhibitors can target multiple kinases in addition to ROS1. For example, entrectinib is a multi-kinase inhibitor that targets not only ROS1 but also ALK, demonstrating a 79.2% overall response rate and 12.0 months of progression-free survival in patients with CNS metastases [44]. However, it is found to be ineffective in cases resistant to crizotinib. The specificity of entrectinib has led to its approval by the FDA as a targeted agent for treating advanced ROS1 rearrangement-positive NSCLC. On the other hand, crizotinib, a first-generation multi-kinase inhibitor, has limitations in its selectivity and coverage of on-target resistance mutations and poor blood-brain barrier penetrance. Next-generation TKIs have shown promise in addressing crizotinib-resistant NSCLC, including repotrectinib for the *G2032R/D2033N* fusion mutation, orlatinib and taletrectinib for the *G2032R* fusion mutation, and cabozantinib and brigatinib for the *CD74* fusion mutation. Notably, repotrectinib has been approved as a first-line agent for ROS1 rearrangement-positive NSCLC, showing 90 times higher efficacy than crizotinib [45].

The management of NSCLC harboring ROS1 alterations has revolutionized the treatment of oncogene-driven cases with TKIs. Despite this progress, the development of acquired resistance poses a substantial obstacle, impeding the broader clinical effectiveness of these targeted therapies. Combination therapy has the potential to enhance efficacy and reduce adverse effects. The application of immune checkpoint inhibitors (ICIs) alone or in conjunction with chemotherapy has been comprehensively investigated in patients with ROS1-rearranged NSCLC [46]. Notably, most ROS1-positive tumors exhibit low PD-L1 expression and have a low tumor mutation burden (TMB). Research into alternative signaling pathways that contribute to therapy resistance in ROS1-positive lung cancer is crucial for developing more effective compounds for combination treatments.

**Table 3:** Inhibitors for ROS1 fusion variants with combination therapy/agent in NSCLC.

Inhibitors for ROS1 Fusion Variants and Combination Therapy	Starting Dose	Stage of Disease	Previous Treatment	Sample Size (Phase)	Objective Response Rate (ORR) and/or Median Progression-Free Survival (PFS)	References
Repotrectinib	160 mg/day	meningeal carcinomatosis (G2032R mutated)	Cisplatin/pemetrexed and Crizotinib	Case report	-	[39]
Entrectinib	600 mg/day	Advanced	Not reported	168 patients	ORR: 68%, 15.7 months	[40]
Crizotinib	250 mg twice per day	Advanced	Not reported	49 patients (two groups: CD74-ROS1 and non–CD74–ROS1)	ORR: 94.11% (non–*CD74*-ROS1) and 73.68% (*CD74*-ROS1), PFS: 17.63 months (non–*CD74*-ROS1) and 12.63 (*CD74*-ROS1)	[41]
				34 patients (phase 2)	ORR: 88.9%, PFS: 16.8 months	[47]
Ensartinib	225 mg/day	Advanced	Chemotherapy	59 patients (phase 2)	ORR: 27%, PFS: 4.6 months	[42]
Taletrectinib	600 mg/day	Advanced (G2032R mutated)	Chemotherapy or Crizotinib	105 patients (phase 2)	ORR: 91.7% (intracranial)	[43]
Ceritinib	750 mg/day	Advanced	Few patients pre-treated with Crizotinib	32 patients (phase 2)	ORR: 62%, PFS: 19.3 months (crizotinib-naive patients)	[48]
Lorlatinib	100 mg/day	Advanced	CNS radiation	16 patients (phase 2)	ORR: 87%, PFS: 38.8 months (intracranial)	[49]

## 3. Clinical Trial Efficacy of Targeted Therapies in NSCLC

Numerous studies in the field of clinical trials have demonstrated the effectiveness of different generations of tyrosine kinase inhibitors (TKIs), such as afatinib [50], alectinib, abemaciclib [51], crizotinib [52], erlotinib [53], gefitinib, lorlatinib, lazertinib [54], and osimertinib [55], in treating advanced NSCLC patients with specific EGFR, ALK, and KRAS mutations. In a global, phase III clinical trial labeled LASER301, the effectiveness of lazertinib was compared against gefitinib in treatment-naïve NSCLC patients with EGFR mutations, specifically those with exon 19 deletion (ex19del) or *L858R* mutations. This trial showed that lazertinib significantly outperformed gefitinib as a first-line treatment for advanced NSCLC patients while displaying a manageable safety profile [54].

Immune checkpoint inhibitors that target programmed cell death 1 (PD-1) or its ligand (PD-L1), either alone or in combination with anti-cytotoxic T-lymphocyte-associated antigen 4 (CTLA-4), have demonstrated effectiveness in treating patients with metastatic NSCLC. In the Phase III NEPTUNE study, researchers investigated the combination of durvalumab, a PD-L1 monoclonal antibody, and tremelimumab, a monoclonal antibody targeting CTLA-4, in Chinese cohorts with PD-L1 TC < 1% population. This exploratory analysis included randomization (1:1) of patients with EGFR and ALK mutations, stratified by PD-L1 tumor cell (TC) expression (≥25% vs. <25%). The results indicated a potential improvement in overall survival (OS) compared to chemotherapy. Notably, the 24-month OS and 12-month PFS rates suggested a benefit in survival outcomes [56]. In the MYSTIC trial, researchers conducted an open-label, phase 3 randomized clinical trial to treat naïve patients without specific EGFR or ALK genetic mutations. The patients were divided into three groups, i.e., one received durvalumab alone, another received durvalumab combined with tremelimumab, and the third received platinum-based doublet chemotherapy. The trial revealed that patients with bTMB ≥ 20 mutations/megabase (a blood tumor mutational burden) had better overall survival with durvalumab plus tremelimumab compared to chemotherapy (21.9 months versus 10.0 months). Additionally, the study found that severe treatment-related side effects were less common with durvalumab alone (14.9% of patients) and durvalumab plus tremelimumab (22.9% of patients) compared to chemotherapy (33.8% of patients) [57].

In another phase III clinical trial, a combination of immune-checkpoint inhibitors, nivolumab (anti-PD-1 antibody) and ipilimumab (CTLA-4 antibody), demonstrated a significant improvement in overall survival and delayed time-to-definitive deterioration compared to chemotherapy. This was observed in the first-line treatment of metastatic NSCLC patients without EGFR or ALK gene mutations [58]. In a randomized phase II clinical trial, researchers investigated the efficacy of combining afatinib (a tyrosine kinase inhibitor) with cetuximab (a monoclonal antibody) in previously untreated patients with EGFR-mutant NSCLC. The results showed that the combination treatment did not improve progression-free survival compared to patients receiving afatinib alone. Additionally, there was no significant difference in the response rate or overall survival between patients treated with afatinib alone and those receiving the combination therapy with cetuximab [50].

Phase III clinical trials have thoroughly assessed the safety and efficacy of these TKIs and PD-L1 monoclonal antibodies [59] in combination with standard-of-care chemotherapy for first-line treatment in NSCLC patients (Table 4).

## 4. PARP Inhibitors: Combination Therapy

PARPs, also known as poly ADP ribose polymerases, are a group of 18 proteins that serve as multifunctional post-translational modification enzymes crucial for a wide range of important cellular functions. These processes encompass the regulation of chromatin structure, DNA damage repair, facilitating DNA replication, regulating gene transcription, and apoptosis. The PARP enzyme family, composed of highly abundant nuclear proteins, is important for DNA repair processes such as base excision repair (BER), homologous recombination repair (HRR), and alternative end joining. These repair pathways are vital for repairing DNA damage caused by various alkylating agents [60].

Among the DNA-dependent PARPs, PARP1, PARP2, and PARP3 play a significant role in DNA damage repair. They initiate poly ADP-ribosylation through their N-terminal binding domain [61]. Specifically, PARP1 is essential for repairing single-strand breaks (SSBs) and is activated when replication forks stall. It achieves this by attaching ADP-ribose units to multiple proteins, enabling the restart of replication forks after DNA damage repair. Clinical trial reports have demonstrated the effectiveness of PARP inhibitors (PARPi) in targeting aggressive cancers such as NSCLC and small cell lung cancer (SCLC). PARP1 inhibitors exert their cytotoxic effect through two mechanisms. First, they trap the enzyme at the site of SSBs by preventing the utilization of NAD+. Second, they inhibit PARylation, thereby preventing PARP from binding to DNA. This results in the formation of PARP-DNA complexes, which lead to the collapse and stalling of replication forks, ultimately causing the conversion of SSBs to double-strand breaks (DSBs) and triggering apoptosis [62]. PARPi exerts cytotoxicity by disrupting the DNA damage response pathway, impeding DNA repair, and leading to the death of tumor cells.

Studies have shown that elevated levels of poly (ADP-ribose) are linked to a low infiltration of CD8+ cytotoxic T lymphocytes (CTLs), indicating that the activity of PARP1 might impact the interaction between cancer and the immune system [63]. Furthermore, pharmacological PARP1 inhibitors have the potential to be effectively combined with immune checkpoint inhibitors that target cytotoxic T-lymphocyte-associated protein 4 (CTLA-4) or the programmed cell death protein 1 (PD-1)/programmed cell death ligand 1 (PD-L1) interaction. This combination has displayed promising results in certain clinical trials (Table 5). Moreover, the role of PARP in repairing platinum-induced adducts has been extensively studied in preclinical and clinical settings.

## 5. Immunotherapy Regimens

Immunotherapy is a form of cancer treatment that is gaining more prominence. It has revolutionized cancer treatment and is now considered the fourth pillar of cancer treatment, along with surgery, chemotherapy, and radiotherapy. Immunotherapy aims to activate a specific type of immune cell, called cytotoxic T lymphocytes (CTL), which can detect and destroy cancer cells. Therapies that target immune checkpoints, such as programmed cell death-1 (PD-1) and CTL-associated antigen-4 (CTLA-4), have been approved for use in a variety of tumor types, including NSCLC [4]. Since CTL responses are diverse, immunotherapy can be effective against metastatic cancers with different genetic profiles, which other treatments like chemotherapy cannot target.

### 5.1. Immune Checkpoint Inhibitors (ICIs)

Tumors have evolved various strategies to evade the immune system. These strategies include making genetic and epigenetic changes, producing immune-suppressing cytokines like IL-10 and transforming growth factor β within the tumor surroundings, and activating pathways that inhibit T-cell function. The immune system is regulated by molecules known as immune checkpoints, which are found on immune cells and help control the level of immune activity [77]. Immune-checkpoint inhibitors (ICIs) are immunotherapy drugs revolutionizing cancer treatment. Immune checkpoint proteins are present on the surface of immune cells that interact with partner proteins in other cells, including some tumor cells. They work by blocking the interaction between immune checkpoint proteins and their partner proteins, which allows T cells to recognize and destroy cancer cells. The interaction between these proteins sends an “off” signal to T cells, which prevents the immune system from attacking cancer cells. By blocking this interaction, ICIs allow T cells to kill cancer cells and improve patient outcomes.

In recent years, numerous studies in molecular oncology have delved into the intricate regulatory mechanisms governing the expression of immune checkpoints. One extensively studied immune checkpoint pathway is PD-1, or programmed cell death protein-1, a coinhibitory receptor on the surface of cells. It gets activated when it binds to PD-L1 or PD-L2 ligands. PD-1, also referred to as CD279, is a member of the CD28 superfamily. It is classified as a type I transmembrane glycoprotein, featuring a single extracellular immunoglobulin variable domain, a transmembrane region, and a cytoplasmic domain that contains both an immunoreceptor tyrosine-based inhibitory motif (ITIM) and an immunoreceptor tyrosine-based switch motif (ITSM) [78]. All T cells express PD-1 when activated, which helps regulate the immune system’s response to tumors and autoimmune diseases. PD-1 is a negative signal that counteracts the immune system’s response to tumor cells by binding with PD-L1 on tumor cells. PD-L1 stops T cells from attacking healthy cells, which some cancer cells produce in high amounts to evade the immune system [79]. The PD-1/PD-L1 pathway is a major adaptive immune resistance (AIR) mechanism that allows tumors to resist the body’s natural immune response.

Researchers have identified several immune checkpoint molecules, such as PD-1/PD-L1, CTLA-4, *LAG-3*, and TIM-3, which regulate immune responses. The development and use of ICIs targeting CTLA4, PD-1, and PD-L1 have transformed cancer treatment. This innovative approach was recognized with the 2018 Nobel Prize for Medicine and Physiology. Despite the considerable number of newly discovered immune checkpoints, the FDA has approved only a limited number of immune checkpoint inhibitors (ICIs) due to the complexity of their mechanisms [77].

As specified in Section 2.1, TKIs are highly recommended for NSCLC patients with EGFR, ALK, and ROS1 genetic rearrangements. For NSCLC patients without treatable mutations, ICIs can be a long-lasting treatment option, particularly for those with high levels of PD-L1 expression (Table 6). Although combination therapies are being used, many patients do not experience clinical benefits from ICIs, prompting ongoing research efforts to enhance their effectiveness.

### 5.2. Immune Checkpoint Co-Inhibitory Receptors

PD-L1 monoclonal antibodies have been increasingly used in the past decade to treat advanced-stage NSCLC. However, not all patients benefit from this therapy due to the development of primary resistance and acquired adaptations. Apart from PD-L1, other immune checkpoints that act as co-inhibitory receptors, like lymphocyte-activation gene-3 (*LAG-3* or *CD223*), T-cell immunoglobulin (Ig), and mucin domain-3 protein (TIM-3), are believed to have putative therapeutic targets. *LAG-3*, a member of the immunoglobulin superfamily, has a similar structure to the CD4 co-receptor, which results in a stronger binding to MHC class II. TIM-3 is an immunoregulatory molecule discovered as an inhibitory molecule on the surface of Th1, Th17, and Tc1 cells, which binds to the ligand galectin-9. In a recent study, Datar et al. [91] analyzed the expression of T-cell markers PD-1, *LAG-3*, and TIM-3 in NSCLC patients. A strong correlation was found among these markers and the production of higher levels of T-cell activation, cytotoxicity, and apoptosis markers. The study utilized multiplexed quantitative immunofluorescence (QIF) to observe the expression of these markers in EGFR-mutated cases. The results demonstrated lower expression of these markers in EGFR-mutated NSCLC patients. *LAG-3* and TIM-3 were detected in tumor-infiltrating lymphocytes (TILs) without PD-1 expression.

### 5.3. Targeted Antibodies

Three main types of targeted antibodies can be used as therapeutic drugs in treating NSCLC, i.e., monoclonal antibodies, antibody-drug conjugates, and bispecific antibodies. They show anti-tumor activity against various targets such as EGFR, vascular endothelial growth factor (VEGF), PD-1, CTLA-4, receptor activator of nuclear factor-kappa B ligand (RANKL), insulin-like growth factor 1 receptor (IGF-1R), human epidermal growth factor receptor (HER2), cMET, and AXL [92]. Monoclonal antibodies (mAb) are a type of treatment that targets specific proteins in the body and are prime examples of personalized therapeutics. Cetuximab, necitumumab, nimotuzumab, and ficlatuzumab all target the EGFR signaling pathway. Bevacizumab and ramucirumab, on the other hand, target the VEGF (vascular endothelial growth factor) pathway. Atezolizumab, durvalumab, nivolumab, sintilimab, and pembrolizumab all work by binding to the PD-1 protein. Ipilimumab and tremelimumab work as anti-CTLA-4 agents. Denosumab is an anti-RANKL treatment, while figitumumab targets the IGF-1R.

Nivolumab [93] and pembrolizumab [83], anti-PD-1 checkpoint inhibitor monoclonal antibodies, have been shown to improve overall survival compared to platinum-based chemotherapy in advanced NSCLC patients (Table 6). Several studies have shown that using ipilimumab [94] and durvalumab [95], along with first-line chemotherapy, does not improve the overall survival rate compared to chemotherapy alone. However, the combination of pembrolizumab [96] and ramucirumab [97] with platinum-based chemotherapy has been shown to improve the outcomes for patients with metastatic NSCLC. Additionally, combining ramucirumab with Erlotinib is an effective therapeutic drug for EGFR mutant NSCLC patients with high PD-L1 expression [98].

Antibody-drug conjugates, or ADCs, humanized or human monoclonal antibodies covalently attached to a cytotoxic drug, are among the fastest-growing anticancer drugs. ADCs, including ado-trastuzumab emtansine, an effective drug harboring HER2 mutations, and telisotuzumab vedotin, are anti-cMET-directed antibodies. Bispecific antibodies are a type of tumor immunotherapy with two binding domains, allowing them to simultaneously target two different types of antigens. Amivantamab (FDA-approved, 2021) is the only bispecific antibody for NSCLC patients, and it targets both EGFR and MET pathways. In a clinical trial conducted by Wang et al. [99], amivantamab was given to six patients with EGFR 20 exon insertion mutation, resulting in a 28.6% overall response rate. However, a conclusion regarding the median progression-free survival was not reached after an 8.7-month follow-up period.

## 6. Nanobodies for Targeted Therapeutic Delivery

Nanobodies (Nbs) are a unique class of smallest antibody fragments (14 kDa) derived from heavy-chain only IgG antibodies present in the serum of camelids. They have only a single antigen-binding domain. In recent years, nanobodies have become promising carriers in drug delivery therapies that show higher biochemical stability, strong tumor penetration, high affinity, and tissue specificity compared to conventional antibodies (IgGs). In the treatment of lung adenocarcinoma, about 50% of patients develop brain metastasis at stage III, and most of the chemotherapeutic drugs are not able to cross the blood-brain barrier [100]. Recently, Kovalchuk et al. [100] found the strong tumor penetration of bispecific anti-VEGF-A/Ang2 nanobody in a mouse model and reported partial meningeal brain metastases in lung adenocarcinoma. In NSCLC, genotypes of some patients did not respond to the monoclonal antibodies due to the overexpression of the epidermal growth factor receptor (EGFR). Tabtimmai et al. [101] proposed a strategy to use humanized-cell penetrable VH/V_H_H Nbs (transbodies) that interfere with tyrosine kinase activity of EGF receptors and reported suppressive activity on the mortality of cancer cells. In this study, researchers used the combination of an in vitro approach and computational modeling and reported that VH/V_H_H transbodies use CDR2 and CDR3 to bind with EGFR domains, which inhibits intracellular signaling by targeting the kinase activity. Moreover, Pham et al. [102] employed Sortase A and OaAEP1 ligases to conjugate extracellular vesicles (EVs) with nanobodies that enhance the delivery uptake of encapsulated paclitaxel drug at low doses by targeting EGFR, HER2, and SIRP alpha receptors. This nanobody therapeutic delivery vehicle approach was further investigated in innate immune response, suppressing tumor growth with increased immune cell infiltration through the RIG-I-like receptor (RLR) pathway. In this study, Peng et al. [103] reported the robust anti-cancer response and immunogenic cell death of NSCLC cells with RIG-I agonist immRNA (an immunomodulatory) using nanobody-mediated human red blood cell extracellular vesicles (RBCEVs) that also further suppresses breast cancer metastasis in the lung. Studies have shown that EVs derived from mesenchymal stem cells (MSCs) have therapeutic effects in reducing inflammation. Among the different types of MSC-EVs, microRNA miR-21-5p has been linked to decreasing lung cell apoptosis by inhibiting PTEN and PDCD4 [104].

Osimertinib is a very effective tyrosine kinase inhibitor (TKI) in NSCLC patients developing EGFR mutations, but some patients resist TKIs due to some EGFR-dependent mechanisms. Guardiola et al. [105] reported the efficacy of anti-EGF nanobodies when combined with EGFR-TKIs, which activated the downstream proteins, decreased the Hes1 expression, and delayed the appearance of drug resistance. Zhang et al. [106] overcame the tumor microenvironment and provided an effective therapeutic modality to manage NSCLC by combining the anti-EGFR Nbs with phototherapy. The targeted nanobody complex enhanced the tumor hypoxia, decimated the primary tumor, and thus inhibited lung metastasis.

As discussed in Section 5, programmed cell death ligand 1 (PD-L1) plays an important role in immune checkpoints and has been approved as an ideal target for diagnosis and treatment in various tumor types, including NSCLC. Recently, Zhu et al. [107] synthesized ^131^I-labeled Nb109 (a nanobody targeting PD-L1) to inhibit tumor growth in NSCLC cells without toxic effects and described the therapeutic efficacy of ^131^I-Nb109 with in vitro and in vivo studies. Moreover, other studies also reported the potential and sensitivity of Nb109 in assessing the PD-L1 expression in NSCLC tumors [108].

## 7. Natural Drug Therapy: Monotherapy or in Combination

Despite the availability of several anticancer drugs/agents, including immunotherapy, chemotherapy, radiotherapy, and DNA-damaging agents, tumors continue to pose a threat to human health. However, the use of monotherapies has resulted in the development of acquired resistance and adverse toxic effects. The biggest obstacle in cancer treatment is the management of lung cancer, which is characterized by secondary drug resistance and dose-limited toxicity. Fortunately, natural drug therapy has provided a solution to the resistance of anticancer drugs and improved disease-specific survival. Natural bioactive compounds from herbal plants regulate cancer stem cells (CSCs) and help control tumor relapses and metastasis. The self-renewal molecular pathways such as WNT, notch, JAK-STAT, and Hippo signaling pathways sustain the CSCs.

Bioactive compounds have emerged as a promising therapeutic compound in the past decade. They are known to suppress cancer stem cells through signaling pathways, reduce tumor sphere, suppress the activation of COX2 and STAT3, activate mitogen-activated protein kinases, inhibit Skp2 and EGF-mediated Akt activation, and induce paraptotic cell death. Corominas-Faja et al. [109] target CSCs with silibinin application with an EGFR inhibitor and eliminate the tumor sphere in NSCLC containing erlotinib resistance. In addition, curcumin drugs with a low dose of cisplatin targeting CSCs cause cell cycle arrest and inhibit the highly migratory subpopulation in NSCLC [110]. Various significant bioactive compounds such as gigantol [111], chrysotoxine [112], dieckol [113], resveratrol oligomers [114], honokiol [115], ginseng [116], silybin [117], silibinin [109], and baicalin [118], betulinic acid [119], vanillin [120], parthenolide [121], atractylenolide III [122], ginsenoside Rh2 [123], etc., are known to target signaling pathways and eliminate cancer cells in NSCLC patients (Table 7).

Combining natural agents with first- or second-line therapy can provide promising candidates to overcome drug resistance and related adverse effects through various molecular mechanisms. A natural sesquiterpene lactone, eupatolide alone, delayed tumor growth in animal studies and enhanced the cytotoxic effects of chemotherapeutic drugs cisplatin and 5-Fluorouracil (5-FU) [124]. Hu et al. [125] also conducted synergistic studies and reported the inhibitory effects of amentoflavone (AMF) on PARP-1 and considerably enhanced carboplatin sensitivity in nude mice. Amentoflavone and derivatives were isolated from *Selaginella moellendorffii* and exerted better selectivity towards PARP-1 than Olaparib. Liu et al. [126] treated NSCLC cell lines with a combination of second-generation EGFR inhibitors—afatinib and vinorelbine, a semi-synthetic vinca alkaloid—in low doses and found a reduction in anti-apoptotic proteins. In future clinical studies, natural products alone or combined therapies can provide safe, novel, and potent chemotherapeutic drugs at less cost.

**Figure 2:**
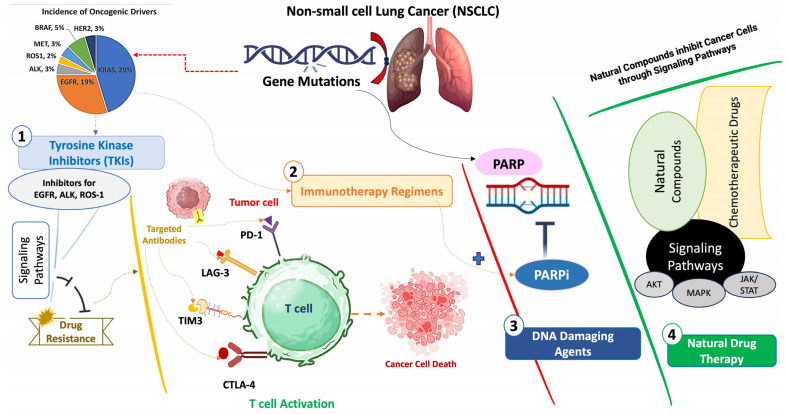
Combinatorial therapeutic strategies: tyrosine kinase inhibitors (TKIs), immunotherapy regimens, DNA-damaging agents, and herbal therapy in NSCLC to combat drug resistance.

Immune checkpoint markers are crucial in diagnosing and treating NSCLC. While combination therapies are widely used, some patients do not experience the desired clinical benefits. Researchers are tirelessly working towards finding ways to improve the efficacy of these therapies. One promising approach is to combine natural agents with targeted therapy. The combinatorial approach has the potential to overcome drug resistance and related adverse effects through various molecular mechanisms, making it a promising candidate for the treatment of NSCLC (Figure 2).

## 8. Conclusions and Future Perspective

The molecular genetics of non-small cell lung cancer is highly complex and heterogeneous. Over the past two decades, researchers have made significant strides in understanding the biological factors contributing to the development of NSCLC. Targeted and personalized medicine has emerged as a promising approach to managing advanced NSCLC and reducing the high failure rates of chemotherapy. Molecular studies have led to the development of novel targets and combination therapies for systemic treatment of NSCLC. Systemic treatments in NSCLC have evolved through molecular research that has identified novel target and combination therapies. Although clinical research in targeted therapies has fundamentally revolutionized the treatment of NSCLC patients, many patients are still non-responders due to the continual development of resistance. Recent immunotherapy advances with immune checkpoint inhibitors alone or combined with tyrosine kinase inhibitors (TKIs), DNA-damaging agents (PARP inhibitors), or chemotherapy are promising for treating advanced lung cancer. In addition, natural drug therapy, such as monotherapy or in combination with chemotherapeutic drugs, has shown promising results in inducing cell cycle arrest through signaling pathways in NSCLC. Future developments in drug-resistant therapies will likely come from targeted nanobodies, combination immunotherapies, and personalized approaches that consider molecular biomarkers.

## Figures and Tables

**Table 2:** ALK tyrosine kinase inhibitors with combination therapy/agent in NSCLC.

Inhibitors for ALK Rearrangements and Combination Therapy	Starting Dose	Stage of Disease	Previous Treatment	Sample Size (Phase)	Objective Response Rate (ORR) and/or Median Progression-Free Survival (PFS)	References
Ceritinib	750 mg/day	Stage IIIB/IV	Untreated	189 patients (phase 3)	PFS: 16.6 months	[29]
Crizotinib	250 mg/day twice daily	Advanced	Untreated	104 patients (phase 3)	ORR: 87.5%, PFS: 11.1 months	[30]
Alectinib	600 mg/day twice daily	Advanced	Pre-treated with chemotherapy and crizotinib	119 patients (phase 3)	ORR: 50.6%, PFS: 10.9 months	[32]
			Untreated	152 patients (phase 3)	ORR: 85.7%	[31]
Lorlatinib	100 mg/day	Advanced	Pre-treated with 1st and 2nd generation TKIs	10 patients (phase 1)	ORR: 64%, PFS: 6.5 months	[33]
Brigatinib	90 mg/day	Stage IIIB or IV	Not reported	20 patients (phase 2)	ORR: 40%, PFS: 7 months	[34]
Alectinib and Bevacizumab	Alectinib (600 mg/day) twice daily and bevacizumab (15 mg/kg) every 3 weeks	Advanced	55% of patients were untreated, and 46% pre-treated with ALK inhibitors	11 patients (phase I/II)	PFS (3 pre-treated patients): 9.5 months	[35]

**Table 4:** Active phase-III clinical trials of targeted combination therapies in NSCLC patients.

Identifier: Clinical Study ID Numbers	NSCLC Patients (Any Mutation or Marker Expression)	Number of Baseline Participants		Treatment Arm (Experimental and Comparator Treatment)	Serious Adverse Events	Primary Outcome Measures: Median (Months) [DFS (Disease-Free Survival); OS (Overall Survival); PFS: (Progression-Free Survival)]
		Female	Male			
NEPTUNE: NCT02542293	EGFR and ALK mutation-positive	256 (26.9%)	697 (73.1%)	Durvalumab + Tremelimumab	Global: 47.07%	bTMB ≥ 20 mut/Mb: OS (11.7)
				SoC Chemotherapy	Global: 28.07%	bTMB ≥ 20 mut/Mb: OS (9.1)
MYSTIC: NCT02453282	EGFR and ALK wild type	346 (30.9%)	772 (69.1%)	Durvalumab Monotherapy	35.50%	PD-L1 (TC ≥ 25%): OS (16.3)
				Durvalumab + Tremelimumab	47.98%	PD-L1 (TC ≥ 25%): OS (11.9), PFS (3.9)
				SoC Chemotherapy	31.82%	PD-L1 (TC ≥ 25%): OS (12.9), PFS (5.4)
PEARL: NCT03003962	EGFR and ALK mutation-positive with PD-L1 high expression	132 (19.7%)	537 (80.3%)	Durvalumab	39.40%	OS (14.6), PD-L1 (TC ≥ 25%): OS (14.6)
				Platinum-based SoC	31.80%	OS (12.8), PD-L1 (TC ≥ 25%): OS (15.0)
NCT02438722	EGFR mutation positive	112 (66.7%)	56 (33.3%)	Afatinib dimaleate + Cetuximab	37.18%	PFS (11.9)
				Afatinib dimaleate	35.29%	PFS (13.4)
LASER301: NCT04248829	EGFR mutation-positive	251 (63.9%)	142 (36.1%)	Lazertinib	26.02%	PFS (20.6)
				Gefitinib	25.89%	PFS (9.7)
RELAY: NCT02411448	EGFR mutation-positive	294 (63.5%)	169 (36.5%)	Ramucirumab + Erlotinib	29.41%	PFS (19.4)
				Placebo + Erlotinib	20.89%	PFS (12.4)
AURA3: NCT02151981	EGFR mutation-positive	269 (64.2%)	150 (35.8%)	Osimertinib	30.11%	PFS (10.1)
				Platinum-based doublet chemotherapy	26.47%	PFS (4.4)
LEAP-007: NCT03829332	PD-L1 (TPS) greater than or equal to 1%	169 (27.1%)	454 (72.9%)	Pembrolizumab + Lenvatinib	56.63%	OS (14.1), PFS (6.6)
				Pembrolizumab + Placebo	33.97%	OS (16.4). PFS (4.2)
NCT03631706	High PD-L1-tumor expression with no EGFR or ALK mutation	78 (25.7%)	226 (74.3%)	M7824	59.60%	OS (21.1), PFS (7.0)
				Pembrolizumab	40.13%	OS (22.2), PFS (11.1)
JUNIPER: NCT02152631	KRAS mutation	181 (40%)	272 (60%)	Abemaciclib	42.26%	OS (7.4)
				Erlotinib	24.57%	OS (7.8)
CodeBreak 200: NCT04303780	KRAS mutation	141 (40.9%)	204 (59.1%)	AMG 510	53.85%	PFS (5.62)
				Docetaxel	44.37%	PFS (4.47)
NCT02838420	ALK mutation-positive	89 (47.6%)	98 (52.4%)	Alectinib	15.20%	-
				Crizotinib	25.81%	PFS (11.1)
ALEX: NCT02075840	ALK mutation-positive	171 (56.4%)	132 (43.6%)	Alectinib	28.95%	-
				Crizotinib	29.80%	PFS (11.1)
NCT03052608	ALK mutation-positive	175 (59.1%)	121 (40.9%)	Lorlatinib monotherapy	34.23%	-
				Crizotinib monotherapy	27.46%	PFS (9.3)
CheckMate 9LA: NCT03215706	-	215 (29.9%)	504 (70.1%)	Nivolumab + Ipilimumab + Chemotherapy	56.70%	OS (14.13)
				Chemotherapy only	41.26%	OS (10.74)
PEARLS: NCT02504372	-	373 (31.7%)	804 (68.3%)	Pembrolizumab	24.48%	DFS (53.8), PD-L1 (TPS ≥ 50%): DFS (67.0)
				Placebo	15.49%	DFS (43.0), PD-L1 (TPS ≥ 50%): DFS (47.6)
POSEIDON: NCT03164616	-	243 (24.0%)	770 (76.0%)	Tremelimumab + Durvalumab + SoC chemotherapy	44.24%	-
				Durvalumab + SoC chemotherapy	40.12%	OS (13.3), PFS (5.5)
				SoC chemotherapy	35.14%	OS (11.7), PFS (4.8)

**Table 5:** Poly (ADP-ribose) polymerase (PARP) inhibitors with combination therapy/agent in NSCLC.

PARP Inhibitor and Combination Therapy/Agent	Dosing	Inhibition Effect	Model/Clinical Trial	References
Talazoparib + Avelumab (immune checkpoint inhibitor)	Talazoparib (1 mg/day) + Avelumab (800 mg every 2 weeks)	Changes in genes related to homologous recombination	Clinical study	[64]
Veliparib + Nivolumab (PD-1) + chemotherapy agents (carboplatin and Paclitaxel)	Veliparib (120 mg twice daily) + Nivolumab (360 mg) + carboplatin (AUC 6 mg/mL·min) + paclitaxel (200 mg/m^2^)	-	Clinical study	[65]
Rucaparib + Olaparib	Rucaparib (25 μM) and Olaparib (40 μM)	Activate cGAS/STING, downstream type I IFN signaling, and CCL5 secretion	Pre-Clinical study	[66]
Rucaparib + KP372-1	Rucaparib (15 µM) and KP372-1 (0.4 µM)	NQO-1-dependent DNA damage	Pre-Clinical study	[67]
Olaparib + Ruthenium [Ru(dppz)_2_(PIP)]^2+^	-	DNA Double-strand break (DSB) and level of reactive oxygen species (ROS) increases	Test on zebrafish embryos	[68]
Olaparib + Gefitinib (EGFR inhibitor)	-	low mRNA expression of CtIP	Phase 2 clinical trial	[69]
Niraparib + Radiation	Niraparib (30 mg/kg) + 8 Gy × 3 radiation	CD8+ T lymphocytes Increase, and the STING/TBK1/IRF3 pathway is activated	In vivo studies on mice	[70]
Talazoparib + Type I PRMT inhibitor: MS023 (epigenetic modulator)	Talazoparib (50 nM) and MS023 (2 μM)	DNA Damage (γ-H2AX foci elevated)	Pre-Clinical study	[71]
Talazoparib + Olaparib + Gemcitabine (Chemotherapeutic drug)	Gemcitabine (80 mg/kg) + Talazoparib (0.333 mg/kg)	Induces single-strand DNA breaks	Xenograft model	[72]
Olaparib + Ceralasertib (ATR inhibitor)	Olaparib (50 mg/kg once daily) + Ceralasertib (12.5 mg/kg twice daily)	Activates ATM-dependent signaling pathway	Xenograft model	[73]
Olaparib + APR-246 (PRIMA-1^Met^)	Olaparib (0–80 μM) and APR-246 (0–40 μM)	ROS production increased, and p53 translocated to the mitochondria	Pre-Clinical study	[74]
Olaparib + Carbon ion (^12^C) radiotherapy	Olaparib (2 μM) + ^12^C ion (0.5 Gy)	Activation of MMP-2,-9 transcription pathway	Pre-Clinical study	[75]
Fluzoparib + Radiotherapy	Fluzoparib (30.84 μM)	Activation of p21 via p53 pathway	Xenograft mouse model	[76]

**Table 6:** Immune checkpoint inhibitors with PD-L1 expression in the treatment of NSCLC.

Therapy	Key Biomarker	Overall Survival Rate	References
Pembrolizumab + datopotamab deruxtecan (Dato-DXd)	PD-L1 (50% expression level)	62%	[80]
Pembrolizumab monotherapy		29.6%	
Pembrolizumab monotherapy	PD-L1 (≥50% expression level)	-	[81]
Nivolumab + ipilimumab	PD-L1 (1% expression level)	62.6%	[82]
Chemotherapy		56.2%	
Pembrolizumab monotherapy	PD-L1 (50% expression level)	80.2%	[83]
Five chemotherapy regimens		72.4%	
Pembrolizumab monotherapy	PD-L1 (1% expression level)	-	[84]
Docetaxel (Chemotherapy)		-	
Nivolumab monotherapy	PD-L1 (more than 5% expression level)	64%	[85]
Docetaxel (Chemotherapy)		-	
Pembrolizumab + pemetrexed (chemotherapy)	PD-L1 (50% expression level)	69.2%	[86]
Placebo combination therapy		49.4%	
Nivolumab + ipilimumab with two cycles of chemotherapy	PD-L1	63%	[87]
Chemotherapy		47%	
Pembrolizumab monotherapy	PD-L1 (50% expression level)	21.8%	[88]
Pembrolizumab monotherapy	PD-L1 (90–100% expression level)	60%	[89]
Sintilimab monotherapy	PD-L1 (1–50% expression level)	88.5%	[90]

**Table 7:** Natural drug therapy targeting various potential mechanisms in NSCLC.

Therapeutic Drugs	Potential Function	Signaling Pathway	Assay/Analysis	References
Curcumin + cisplatin	Cell cycle arrests (downregulation of cyclin D1), p21 expression increases, and the activation of Apaf1 and caspase-9	Intrinsic apoptotic pathway	Apoptosis and migration assay	[110]
Resveratrol oligomers: α-viniferin + ε-viniferin (Origin: *Vitis* sp.)	p-AKT expression decreases, and cleaved PARP expression increases	Akt pathway	MTT assay, flow cytometric assay, immunofluorescence assay, colony formation assay, animal experimentation, and TUNEL assay	[114]
Bibenzyl: Gigantol (Origin: *Dendrobium draconis*)	Down-regulation of caveolin-1 (Cav-1), activation of Cdc42	Cav-1-dependent pathway	Apoptosis and invasion assay and Western blot analysis	[111]
Flavonoid: Baicalin	E-cadherin increases and vimentin decreases; p-PDK1 and p-AKT levels decrease	PDK1/AKT signaling pathway	Immunofluorescence, Western blot, and immunohistochemistry assay	[118]
Bibenzyl: Chrysotoxine (Origin: *Dendrobium draconis*)	Suppression of Sox2	Src-Akt pathway	Spheroids formation assay, WST assay, and Western blot analysis	[112]
Vanillin (Origin: *Vanilla planifolia*)	Downregulation of Oct4 and Nanog	Ubiquitin-proteasomal pathway	Anchorage-independent growth assay, spheroid formation assay, Western blot analysis, ad immunoprecipitation assay	[120]
Polyphenol: Silibinin + erlotinib	ALDH cells decrease	KEGG pathway	Microarray, ALDEFLUOR activity assay, and tumor sphere formation assay	[109]
Sesquiterpene lactone: Parthenolide (origin: *Tanacetum parthenium*)	Inhibition of B-Raf and c-Myc	MAPK/Erk pathway	Clone formation assay, flow cytometry, Western blot, and immunohistochemistry assay	[121]
Polyphenol: Dieckol, (origin: *Ecklonia cava*)	Activates E-cadherin	P13K/AKT/mTOR signaling pathway	MTT assay, flow cytometry, and immunoblotting technique	[113]
Flavonoid: Silybin (origin: *Silybum marianum*)	Inhibition of Skp2 and EGF-mediated Akt activation	Skp2/p27 pathway	Cell viability and colony formation assays, cellular thermal shift assays, Western blotting, molecular docking, and animal studies	[117]
Lactones: Atractylenolide III (ATLIII) (origin: *Atractylodes chinensis*)	IFN-γ-induced indoleamin-2,3-dioxygenase-1 (IDO) expression	Jak3 and Stat3 pathway	Flow cytometry, Western blot analysis, immunofluorescence assay, promoter luciferase assay, CHIP assay, site-directed mutations, and in vivo studies	[122]
Ginsenoside Rh2 (origin: Ginseng)	Decreased the protein level of matrix metalloproteinases (MMPs) and vascular endothelial growth factor (VEGF), which prevents metastasis	-	Flow cytometry, cell proliferation, wound healing assay, enzyme-linked immunosorbent assay (ELISA), Western blot analysis, RT-qPCR, immunohistochemistry, and animal studies	[123]
Pentacyclic triterpenoid: Betulinic acid + ERK inhibitor (U0126, trametinib) + Hydroxychloroquine	Protective Autophagy and activation of mitogen-activated protein kinases	AKT pathway	Cell viability assays, siRNA transfection, flow cytometry, Western blot analysis, mRFP-GFP-LC3 adenovirus transfection, transmission electron microscopy (TEM), and in vivo studies	[119]
Biphenolic: Honokiol (origin: *Magnolia*) combined with Paclitaxel	Induces paraptotic cell death	MAPK pathway	Cell viability assays, clonogenic assay, transmission electron microscopy (TEM), in vivo tumor growth inhibition assay, immunofluorescence staining, siRNA transfection, flow cytometry, Western blot analysis, and measurement of intracellular and mitochondrial Ca^2+^	[115]
Ginseng Rg3 combined with chemotherapy	Myelosuppression decreased	-	Randomized double-blind trial with III-IV NSCLC 414 patients	[116]
Sesquiterpene lactone: Eupatolide (origin: *Inula helenium*) Eupatolide + Cisplatin and Eupatolide + 5-FU	Suppresses the activation of STAT3	Stat3 pathway	Cell proliferation and viability, xenograft study, Western blot, qRT-PCR analysis, and cell apoptosis	[124]
Biflavonoid: Amentoflavone (origin: *Selaginella moellendorffii*) Amentoflavone + carboplatin	Inhibits PARP-1; the number of apoptotic cells in tumor tissues increases	Mitochondrial apoptotic pathways	Intracellular PAR assay, PARP-1/2 inhibition assay, tunnel assay, transfection for PARP-1 silencing, Western blot analysis, immunofluorescence histochemistry, and xenograft experiments	[125]

## Data Availability

No new data were created or analyzed in this study.
